# Therapeutic failure and eventual solution for skin necrosis and exposed tendon of the dorsum of the foot: A case report

**DOI:** 10.1002/ccr3.1697

**Published:** 2018-07-01

**Authors:** Yoichi Toyoshima, Toshio Maeda, Takeshi Kijima, Osamu Namiki, Tetsuya Nemoto, Katsunori Inagaki

**Affiliations:** ^1^ Department of Orthopaedic Surgery Showa University School of Medicine Shinagawa‐ku, Tokyo Japan

**Keywords:** dorsum of the foot, foot necrosis, friable skin, microvascular flap, treatment failure

## Abstract

For the treatment of skin necrosis with exposed tendons in rheumatoid arthritis (RA) foot, we should perform microvascular free flap surgery at an early stage without conservative treatment considering the increased risk of infection and the decreased physical activity.

## 1 INTRODUCTION

Delayed wound healing of dorsal foot skin necrosis can be problematic for patients.^1^ The aim of wound treatment is to cover the bone and joint, ensuring that the necrosis is fully covered. The tendon should be covered as fully as possible and treated with free tissue transfer.^2^, ^3^ We performed an extensor digitorum brevis (EDB) flap for the skin necrosis in which the primary skin defect was covered, but the patient had a new skin necrosis at the donor site of the local flap.^4^ The patient was subsequently treated for donor site skin necrosis via negative‐pressure wound therapy (NPWT). Through several therapeutic failures, we sought to determine the appropriate treatment for dorsal foot skin necrosis with exposed tendon.

## 2 CASE REPORT

**Figure 1:**
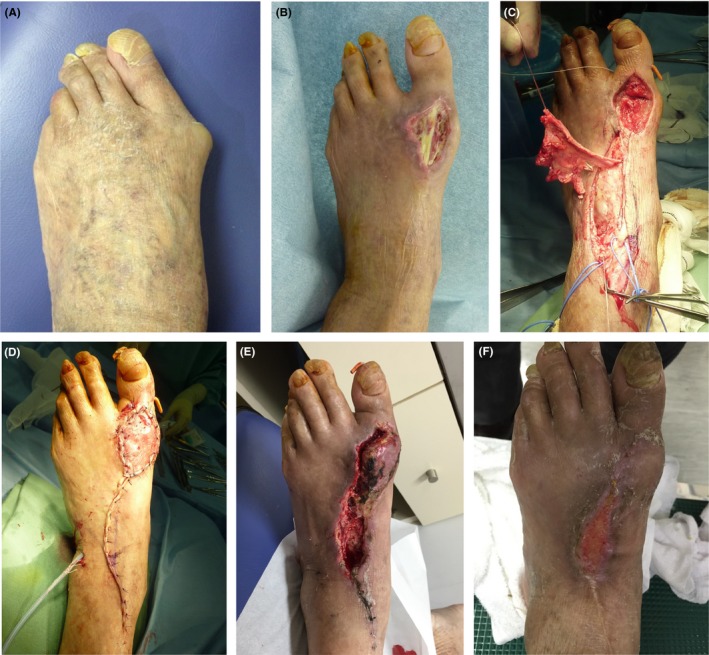
Photographs of the foot. A, Preoperative left foot photograph. Patient had been suffering from rheumatoid arthritis for an extended period and had friable skin. B, Three weeks after surgery, the wound shows maceration upon suture removal. The extensor hallucis longus tendon is still exposed after NPWT. C, D, An extensor digitorum brevis flap was grafted. The site where the EHL was exposed was covered with the flap. E, The skin necrosis occurred at the margin of the lateral donor site without exposed tendon. We performed closure via topical negative‐pressure wound therapy. F, Four months after perforator flap inset, the wound had closed

A 78‐year‐old man suffered from rheumatoid arthritis (RA) (stage IV, class 2) for about 24 years. He had never undergone surgery on his extremities. He was administered methotrexate (4 mg/wk), oral corticosteroids (4 mg/d), and iguratimod (25 mg/d) and had a high course of disease activity (DAS 28‐ESR 4.63). He had pulmonary emphysema and pulmonary fibrosis, as well as chronic kidney failure. In early 2014, he experienced foot pain while walking (Figure 1A).

**Figure 2:**
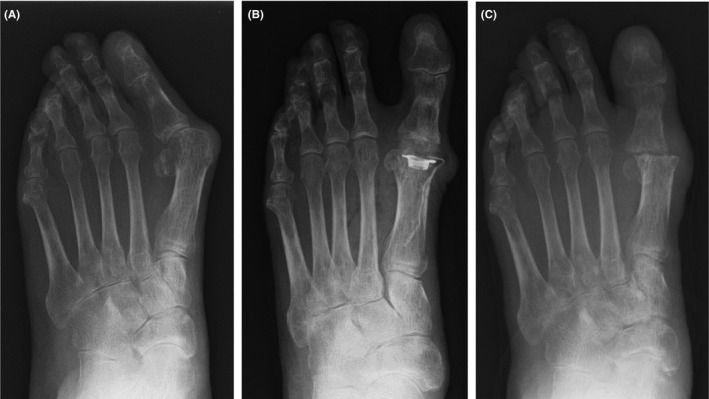
Anteroposterior foot radiographs. A, Patient had pronounced toe deformation. B, After Swanson arthroplasty, deformity of the hallux was corrected. C, At final examination, after removal implant, the hallux metatarsophalangeal joints demonstrated fibrous union

In X‐ray findings, significant deformations of both sides of the great toe were observed (Figure 2A). The patient had an ulcer inside the left metatarsophalangeal joint (MTP). The hallux valgus angle was 52° on the right and 49° on the left. The M1M2 angle was 23° on the right and 18° on the left. The patient required treatment and did not wish to undergo arthrodesis because his job necessitated squatting. Therefore, he had undergone bilateral Swanson implant arthroplasty for the MTP joint of the great toe in October 2014 (Figure 2B).

We made a straight incision at the dorsal MTP joint. We expanded the joint capsule to avoid the inward extensor hallucis longus (EHL) tendon. Articular cartilage of the great toe was invaded by synovitis of rheumatoid arthritis. We closed the skin without significant problems. The patient began indoor walking with full weight‐bearing 1 week after surgery. All stitches were removed 14 days after surgery. The wound of the right foot presented no issues, but we found that the wound of the left foot was macerated, and the EHL tendon was exposed from the wound site. The wound was 3 × 4 cm in size. NPWT was performed on the wound site on the same day, but the wound did not close. Three weeks later, secondary wound closure was attempted and the implant was removed. We immobilized the MTP joint by K‐wire; however, the EHL tendon remained exposed (Figure 1B). By the end of October 2014 (1 month postsurgery), an extensor digitorum brevis (EDB) flap was used to cover skin necrosis (Figure 1C,D). Bone and exposed tendon were sufficiently covered with this flap. However, new skin necrosis occurred at the margin of the lateral donor site without the exposed extensor (Figure 1E). Topical NPWT was performed, and good granulation was observed for 2 weeks. The patient spent 6 weeks to heal the first skin defect and spent more 4 weeks to treat the donor site necrosis. Throughout the treatment period, no pathogenic bacteria were detected in laboratory cultures. In February 2015, at the final examination, the wound had closed (Figure 1F and 2C).

## 3 DISCUSSION

There were some therapeutic failures in this case that caused the skin necrosis with exposed tendon at the first surgery and caused a new skin necrosis at the donor site after the local flap, which prolonged the treatment period. Treatment failure occurred due to the delayed decision of the appropriate flap to use and insufficient consideration of the complications associated with EDB flap and friable skin management at the primary surgery.

Skin necrosis with exposed tendon often requires challenging treatment. First, we used NPWT, which has high initial success with skin surgeries^5^; however, granulation over the tendon was poor, resulting in the need for flap surgery after several weeks. Few useful wound‐covering methods exist for the dorsum site of the foot due to the limited amount of soft tissue.^2^, ^3^ Previous research has suggested that treating with free flap (free abdominal muscle penetrating slit flap, anterolateral thigh free flap, peroneal free flap, scapular free flap) is useful for covering skin defects at the dorsum of the foot.^6^, ^7^, ^8^, ^9^ Free flap requires trained microvascular surgeons and specialized flap monitoring; therefore, we chose EDB retrograde flap, which is relatively simple and the flap is covered with a pedicle.^4^, ^7^, ^8^, ^10^ The advantage to this flap is that collection is inconspicuous, ensuring little dysfunction, and little protrusion of the graft site.^4^ However, the blood supply to the dorsum skin of the foot flows from lateral to medial; therefore, donor site necrosis may occur.^11^ We should have made an inside incision to avoid this complication. The primary skin defect was covered, but the patient had a prolonged treatment and recovery period due to new skin necrosis at the donor site. Considering the increased risk of infection and reduced physical activity associated with the long‐term treatment period, we should have performed a microvascular free flap positively.^12^, ^13^

When multiple joint disorders are present with RA, the presence of friable skin is a problem. The most common complication after foot surgery is “wound dehiscence,” which is a rupture along the suture line of the foot during surgery. Risk factors are age >65 years, steroid use, friable skin, diabetes mellitus, smoking, and systematic factors (eg, sex, stress, medication, obesity, nutrition, and alcohol).^1^, ^14^ Previous research indicated that MTP dorsal deformity, surgical times, and rheumatoid nodules were associated with wound healing.^15^, ^16^ In this case, there was poor skin extensibility at the dorsal part of the foot due to long RA disease duration and steroid use. We should have chosen arthrodesis with small incision without focusing on joint function preservation.

## 4 CONCLUSION

Conservative treatment of dorsal foot skin necrosis with exposed tendon involves a lengthy time period. Considering the increased risk of infection and decreased physical activity associated, we should perform microvascular free flap surgery at early stage without conservative treatment. A comprehensive preoperative surgical plan including available surgical methods, incisions, and rest period should be considered in RA patients with fragile skin.

## CONFLICT OF INTEREST

None declared.

## AUTHORSHIP

YT: involved in conception and design of this case report, drafting of the article, and critical revision of the article for important intellectual content. TM: involved in collection and assembly of case. TK: involved in collection and assembly of case. ON: involved in critical revision of the article for important intellectual content. TN: involved in collection and assembly of case. KI: involved in critical revision of the article for important intellectual content and final approval of the article.

## PATIENT CONSENT

The patient gave consent in writing for data concerning this case to be submitted for publication.
